# Healthier Traditional Green Natural *Aloreña de Málaga* Table Olives Through Mineral Chlorides Fortification During Packaging

**DOI:** 10.3390/foods13244061

**Published:** 2024-12-16

**Authors:** Antonio López-López, José María Moreno-Baquero, Antonio Garrido-Fernández

**Affiliations:** Instituto de la Grasa (IG), CSIC, Campus Universitario Pablo de Olavide, Edificio 46, Ctra. Utrera km 1, 41013 Sevilla, Spain; jose.moreno.baquero@gmail.com (J.M.M.-B.); agarrido@ig.csic.es (A.G.-F.)

**Keywords:** mineral fortified products, potassium, calcium, magnesium, green natural olives

## Abstract

Table olive processing implies losses of mineral nutrients and increased sodium levels due to using brine during fermentation and storage. This study investigated fortifying traditional *Aloreña de Málaga* table olives with mixtures of KCl, CaCl_2_, and MgCl_2_ during packaging to enhance mineral content while reducing NaCl. This research analyses the distribution of cations between olives and brines and employed the Response Surface Methodology (RSM) to model mineral content and their contributions to the Reference Daily Intake (RDI). These models also facilitated the identification of optimal salt combinations for specific goals. Potassium, calcium, and magnesium contents in the olives increased from 657 mg/kg pulp (traditional) to 2578–6349 mg/kg pulp (experimental), from 858 mg/kg pulp to 858–5801 mg/kg, and from 41 mg/kg pulp to 41–2010 mg/kg pulp, respectively. Meanwhile, sodium decreased markedly, from 11,915 mg/kg pulp to about 6665 mg/kg. These changes represent a substantial improvement in the nutritional profile of these olives. Additionally, Principal Component Analysis (PCA) and clustering techniques were used to group treatments based on their mineral nutrient profiles, facilitating the selection of formulations for industrial application. These findings promote the development of nutritionally enriched natural table olives, processed without lye treatment and washing.

## 1. Introduction

Olive fruit is naturally bitter and unpalatable when freshly picked from the tree. One of the earliest methods for reducing bitterness and making olives suitable for human consumption was immersion in seawater or brine, sometimes combined with cracking the olives to accelerate the process and adding seasonings. This method quickly spread throughout the Mediterranean basin with the expansion of the Roman Empire and was fully documented in Columella’s *De Re Rustica on Agriculture* [1]. Additionally, dry salting, a technique common for preserving other foods, was also employed for olives. Both of these preservation methods remain in use at the family level among growing farmers and villages around the Mediterranean Sea. Traditional practices in Greece or Turkey, where olives are preserved in brine or solid salt, represent a continuation and scaling up of these ancient techniques. Developing new approaches, such as the Spanish and California styles, which introduced the application of lye treatment for removing the natural olive bitterness and darkened the fruits, has further expanded global table olive consumption [2]. By the 2021/2022 season, table olive production reached approximately 3.1 million tons [3].

Apart from the well-known traditional Greek or Turkish processes, other regional olive processing methods persist, and the IOC classifies them under the heading of Specialties [4]. One example is the seasoned cracked green *Aloreña de Málaga* table olives, cultivated in the Guadalhorce Valley (Málaga, Spain). The *Aloreña* cultivar is considered an adaptation of *Manzanilla* to the local mountainous environment of this region. In recent decades, these olives have expanded across Spain due to their distinct physicochemical properties, such as their green colour, soft texture, easy pit removal, and sensory attributes, including mild bitterness and well-balanced seasoning. There are three commercial presentations distinguishable by their processing methods and surface colour: fresh *Aloreña de Málaga*, traditional style, and cured olives [5]. The first two are cracked upon arrival at the processing facility, a practice that accelerates debittering, reduces sugar content, and helps preserve the original green colour for longer. These olives are brined in 250 kg plastic drums and stored in cold rooms (to retain the green colour longer) or under shaded plastic covers. The “Fresh” designation applies to olives packaged during the season or while their green colour remains intact. Conversely, olives with a slightly faded green or yellowish tint are commercialised as “Traditional style”. The third option, “Cured” *Aloreña de Málaga,* is stored in ten-ton fermenters similar to Spanish-style processing. These olives usually undergo partial lactic fermentation, transforming their appearance from green to yellow-brown. These olives are then cracked before packaging. Regardless of the presentation, the storage brine initially contains 10–12% NaCl, and the final product has a sodium content of approximately 1.67 g/100 g pulp [6]. Readers are referred to specialised publications for a detailed description of these olives’ different presentation processing methods [5]. Our study specifically focuses on the traditional *Aloreña de Málaga* olives. Like other olive processes, their preservation depends on a sufficient concentration of NaCl in the storage and packaging brines.

Salt is widely used in food preservation to prevent the development of pathogenic and spoilage microorganisms [7]. However, it serves several additional technological purposes, including preventing softening or enhancing flavour. In natural table olives, salt is crucial in mitigating bitterness [2] and modulating the fermentation process, as most lactic acid bacteria struggle to grow at NaCl concentrations above 5% [8]. In the case of *Aloreña de Málaga* olives, valued for maintaining their green colour, lactic fermentation is not favourable. To prevent it, in addition to using salt, the brined olives are often stored in cold rooms [5]. Despite the relatively high salt concentration level in storing “Cured” olives, lactic acid fermentation develops as the temperature rises in spring. This occurs because mixed bacterial cultures, commonly found in these processes, tend to be more resilient and show a wider range of responses than monocultures [8].

Salt intake is a biological imperative, deeply integrated into human physiology, society, and global culture. However, excessive salt ingestion is connected to high hypertension problems, strongly related to cardiovascular morbidity and mortality, with an estimated ~5 million deaths annually. Animal researchers have identified several mechanisms by which high salt intake damages the kidney, brain, vasculature, and immune system, though many potential therapeutic interventions remain untested. While individual salt reduction can lower hypertension, ‘hidden’ salt in industrially prepared foods makes it hard for most people to control their consumption. This problem is further exacerbated by the current food system, which hinders sustained individual control over dietary salt ingestion. There is no unique approach to reducing salt consumption, but it should include the food industry and public legislation initiatives, cultural food habits, and public information [9].

Health agencies have long warned consumers about the negative effects of sugars, saturated fat, and salt on health [10]. Excessive salt consumption remains a major concern, with the World Health Organization [11] recommending a daily intake below 5 g/day for adults. Reducing salt intake is especially beneficial in managing high blood pressure, a key factor in preventing cardiovascular and coronary diseases [12].

A recent study of consumer attitudes towards reduced-salt green table olives, specifically the *Chalkidiki* Greek cultivar, found significant interest in products with lower salt content. Most participants expressed willingness to purchase such products, suggesting that reduced-salt table olives could be a viable strategy for reducing salt intake. This highlights the potential for developing new reduced-salt olive products to meet the growing consumer demand for healthier options [13].

Simple salt reduction during the fermentation, storage, or packaging of table olives poses marked risks of spoilage or safety concerns. Sodium chloride has proven indispensable for effectively managing these risks [2]. Efforts to reduce salt in table olives have primarily relied on substituting sodium chloride with other chloride salts to mitigate such challenges.

For example, potassium and calcium chloride mixtures (0–4% and 0–6%) in Spanish-style *Gordal* olives resulted in suitable pH and titratable acidity profiles. These conditions supported lactic acid bacteria growth and significantly increased potassium and calcium levels while achieving reduced sodium contents [14]. Similarly, in *Manzanilla* olives, CaCl_2_ caused a more rapid pH decrease, while KCl led to lower titratable acidity and higher combined acidity as their concentrations increased. The final products were enriched with potassium and calcium, while sodium levels were reduced to less than half the typical concentration, particularly when the initial NaCl was as low as 4% [15].

In Greece, natural black *Conservolea* olives were fermented using five combinations of sodium, potassium, and calcium chloride salts. These combinations supported vigorous lactic fermentation, achieving high titratable acidity and low pH, although final mineral content was not assessed [16]. In Italy, *Castelvetrano* olives following the Spanish-style or local technique were fermented in brines with salt partially substituted with KCl; the products retained their main bioactive molecules [17]. Similarly, Turkish natural black *Gemlik* olives fermented with binary or ternary salt mixtures, including NaCl, KCl, or CaCl_2_, exhibited titratable acidity up to 4.54 g/L, with lactic acid bacteria and yeast populations reaching 6.51 and 8.26 log CFU/mL, respectively.

Despite these advances, salt reduction during fermentation presents drawbacks, including increased pathogen survival/growth risks, flavour alterations, and potential economic losses [18]. Moreover, post-fermentation operations such as grading, pitting or stuffing, or replacing brine (particularly for glass containers) further complicate salt reduction efforts. These challenges have hindered the adoption of such modifications. Our proposal for fortifying table olives while reducing sodium during packaging avoids these risks by preserving current debittering and fermentation technologies. This approach limits added chloride salts to only those essential for achieving desired levels in the final products. Moreover, with current packaging technologies, including pasteurisation, salt levels can be reduced to Good Manufacturing Practice levels, provided the pH remains below 4.6. This method minimises adverse impacts on organoleptic characteristics, reduces costs and offers a more practical and attractive solution compared to previous approaches.

This work aimed to evaluate the impact of potassium (K), calcium (Ca), and magnesium (Mg) fortification combined with a 50% reduction in sodium (Na) in the packaging brine of traditional *Aloreña de Málaga* olives, with a focus on key characteristics (pH, moisture, and firmness) and the mineral (macro and micro) nutrient content of the final product. The experiment was designed and analysed using the Response Surface Methodology (RSM) to effectively model and interpret the results. The developed models predict the concentrations of mineral nutrients under specific combinations of KCl, CaCl_2_, and MgCl_2_ and estimate their contributions to Daily Reference Intakes. They can also determine the optimal proportions of fortifying salts, considering predetermined objectives for dependent variables such as pH, moisture, firmness, and mineral concentrations.

## 2. Materials and Methods

### 2.1. Olives

The cracked green traditional *Aloreña de Málaga* olives were supplied by Aceitunas Bravo S.L (Alhaurín el Grande, Málaga, Spain) and processed by washing, cracking, and brining in 200 kg plastic drums with an initial 10% NaCl solution. These drums were stored for 20 days in a cold room at 7 °C ± 2 °C. Then, olives followed similar processing steps as the rest of the production.

To perform the experiment, 42 kg of fruits (approximately 220 olives per kg) and 21 L of brine were sent to the pilot plant of the Instituto de la Grasa (IG), CSIC (Sevilla, Spain). The lot was maintained in a cold room at 7 °C ± 2 °C until further use. The brine had a pH of 4.32 and a salt concentration of 7.68% NaCl.

For packaging, the olives first underwent a desalting process at 7 °C ± 1 °C to reduce the salt content to the target of 2.5% NaCl in pulp moisture. This objective was achieved by immersing 42.4 kg of olives in 45.2 L tap water for 48 h, a duration determined by previous desalting experiments to achieve equilibrium. Before packaging, proportional samples of olives and brine were taken to ensure the target salt level had been reached. The desalted fruits had 83.07 g pulp/100 g of olives and 61.24 g moisture/100 g in pulp.

### 2.2. Packaging

The reduced-sodium olives were packaged in 1.2 kg polyethylene terephthalate (PET) containers, each containing 800 g of olives, seasoning material (thyme, fennel, bell pepper, and garlic were added at approximately 2% or 16 g), and 400 mL of brine. Several brine formulations were used, as shown in Table 1. The experiment followed a D-optimum design based on a Simplex lattice, allowing for the estimation of up to cubic terms. The brines were composed of a mixture of KCl, MgCl_2_, and CaCl_2_, with their total concentrations limited to 25 g/L in the packaged product equilibrium. The brine levels in Table 1 were adjusted to account for the olive-to-brine ratio and the hydration degree of salts. The experiment was planned using Design Expert 13.0 (Stat-Easy Inc., Minneapolis, MN, USA).

The brines, irrespective of the other salts, included 2.5% NaCl to ensure equilibrium with the salt concentration in the desalted olive pulp moisture. Thus, the total salt concentration in the brines was maintained at 5 g/100 mL. In addition, the packaging brines were supplemented with common preservatives and additives: 0.1% sodium benzoate, 0.064% ascorbic acid, 0.20% citric acid, 0.1% lactic acid, and 0.20% potassium sorbate.

A control treatment using the same above-mentioned packaging protocols, except the added chloride salts but using 5% salt, was included to serve as a benchmark for comparing the experimental treatments. All containers were stored in a cold room at 7 °C ± 2 °C for two months to allow equilibrium and ensure product preservation.

### 2.3. Physicochemical Characterisation of Brines

Moisture content was determined by drying a sample of olive pulp on stainless-steel plates to constant weight, using a Selecta electric oven (Dry-Big 2002970, Abrera, Barcelona, Spain) maintained at 106 °C.

Instrumental *firmness* was assessed with a Kramer shear compression cell attached to a universal testing machine (Instron, Canton, MA, USA). The cross-head speed was set to 200 mm/min, and firmness values were averaged based on 20 individual measurements of pitted fruit. Shear compression force was expressed as N/g olive pulp [2].

### 2.4. Mineral Composition Analysis in Pulp and Brines

All glassware was soaked overnight in 6 N HCl and thoroughly rinsed with distilled deionised water several times. Wet mineralisation was applied [19] for mineral determination. Briefly, 2.5 g of olive pulp was placed into a 50 mL screw cap Pyrex bottle and heated at 100 °C to constant weight to remove moisture. For brines, 20–25 mL were transferred into similar containers, reducing the solution to 10–15 mL.

Regardless of the sample origin, digestion was performed by adding 5 mL of 65% HNO_3_ to the bottles, which were then heated at 180–220 °C in a sand heater (Selecta Combiplac-Sand 2 6000709, Barcelona, Spain) until grey fumes dissipated and the solution became clear. Following this, 5 mL of a mixture composed of HNO_3_ (65%): HClO_4_ (60%) (1:4) was introduced, and the tubes were heated further until the brown/black fumes vanished, leaving a transparent and colourless solution. After cooling, the solutions were shifted to 25 mL volumetric flasks. The bottles were rinsed with double-deionised water, and the rinsate was added to the flasks. Finally, the flasks were filled to the mark with double-deionised water (Sigma, St. Louis, MO, USA).

Mineral nutrients in the flask solutions were determined by Atomic Absorption (AA) using an air–acetylene flame in a GBC model 932 AA (Victoria, Australia) atomic absorption spectrometer equipped with hollow multi-element cathode lamps for Cu, Cr, Co, Fe, Mn, and Ni (GBC, Victoria, Australia), Na and K (Photron, Victoria, Australia), and Ca and Mg (Photron, Victoria, Australia). To prevent interferences, 2000–5000 µg/mL lantan (La^+3^) was added for Ca and Mg determinations, and 2000 µg/mL (1000 µg/mL) of K (or Na) was added for Na (or K) analysis, to avoid sodium and potassium ionisation.

Phosphorous analysis was conducted using the spectro-colourimetric method outlined in AOAC nº 970.39 [20]. This method relies on the formation of a yellow complex when phosphate, vanadate, and molybdate react in a strongly acidic medium [21].

The 6N hydrochloric acid solution was prepared by diluting concentrated HCl from Fluka (Buchs, Switzerland). Ca, Na, and K stock solutions were obtained from Sigma Aldrich (St. Louis, MO, USA) and PACISA (Madrid, Spain). All reagents were of analytical grade (Panreac, Barcelona, Spain). Further procedural details can be found in López, García, and Garrido [6].

### 2.5. Modelling the Impact of the Added Chloride Salt on Product Characteristics

The levels of KCl, CaCl_2_, and MgCl_2_ salts used in the design were linked to the responses such as firmness, moisture, distribution coefficients (*K_d_*) [22], mineral content in the pulp (regardless of the origin, added or initially present in the provided olives), and their contributions to the reference daily intake. These relationships were studied with the Response Surface Methodology, until special cubic regression models, which, in the canonical (Sheffé) form, were expressed as follows:

(1) $$R=\beta_{0}+\sum_{i=1}^{n}\beta_{i}x_{i}+\sum_{1\leq i<j}^{n}\beta_{ij}x_{i}x_{j}+\sum_{1\leq i<j<k}^{n}\beta_{ijk}x_{i}x_{j}x_{k}+\in$$

In the equation, the response variable R represents the parameters to be studied while the coefficients *βs* are the coefficients to be derived. The ∈ stands for the error [23]. A sequential sum-of-squares ANOVA was performed for each response variable to assess if a more complex model would improve the fit. Variable selection was based on a background option, with criteria set at *p* ≤ 0.01 for inclusion and *p* > 0.1 for exclusion. Models were deemed statistically significant at *p* ≤ 0.05, with an insignificant lack of fit (*p* > 0.05).

When significant interactions were identified, lower-order terms were retained to ensure hierarchical models. This approach guarantees that the models are scale-independent and can be interpreted in actual units. The experiment included three replicates, which allowed for the estimation of pure error and lack of fit. Results were visualised using surface plots in the simplex, with contour lines illustrating various levels of R. The advantages of using RSM include a reduced number of experiments compared to full-factorial designs, leading to lower resource consumption, simultaneous optimisation of multiple factors, the ability to capture non-linear effects and identify optimal combinations, the generation of contour plots and response surfaces that simplify result interpretation, and the capability to perform optimisation with constraints.

Additionally, mineral content data from the olive pulp were subjected to multivariate analysis to identify similar profiles, which could assist producers in selecting specific treatments based on consumer preferences.

Additionally, mineral content tables from olive pulp underwent multivariate analysis to identify comparable profiles, aiding industries in selecting particular conditions based on market preferences.

### 2.6. Statistical Software

Design Expert v.13.0 (Stat-Easy Inc., Minneapolis, MN, USA) was used to design the experiment and analyse responses. XLSTAT v. 2017 (Addisonsoft, Paris, France) was used for *K_d_* estimations and the multivariate analysis. The multivariate analysis used MULTIBIPLOT Version 16.430.0.0 [24] and XLSTAT v 2017 (Stat-Soft, Paris, France).

## 3. Results

### 3.1. Influence of Desalting on Mineral Concentrations in Natural Aloreña de Málaga Olives

Fortifying mineral nutrients requires a desalting step for the stored olives. Initially, sodium concentration in the fruits was 18.6 g/kg pulp and 29.5 g/kg in the brine, but desalting reduced sodium in the pulp to 7.2 g/kg, a 61% reduction (Table 2). Desalting also impacted other minerals naturally present in the olive pulp. Potassium decreased from 2.5 g/kg to 0.8 g/kg (66% reduction), magnesium from 126 mg/kg to 57 mg/kg (55%), phosphorous (P) from 189 mg/kg to 119 mg/kg (37%), manganese (Mn) from 1.04 mg/kg to 0.52 mg/kg (50%), and zinc (Zn) from 4.03 mg/kg to 2.57 mg/kg (36%). However, calcium showed a smaller reduction, from 1.2 g/kg pulp to 1.0 g/kg (13%), as did iron (Fe), from 5.55 mg/kg to 4.04 mg/kg (27%), and copper (Cu), from 3.31 mg/kg to 2.65 mg/kg (20%) (Table 2). The desalting process in directly brined olives resulted in sensible mineral nutrient losses. While potassium, calcium, and magnesium can be replenished during packaging with fortifying brines, micronutrient losses are irreversible and should be carefully considered when applying the fortification methods proposed in this work.

### 3.2. Mineral Concentrations in Olive Pulp and Brine According to Treatments and Their Relationships 

Table 3 and Table 4 present the concentrations of the macro- and micronutrients in the olive pulp and brines following the experimental packaging design for *Aloreña de Málaga* table olives. Besides the expected increase in the fortifying elements, all treatments showed a notable reduction in sodium content, with levels decreasing from 11.9 g/kg to 6.6 g/kg in the pulp (a 44% reduction). This significantly reduces sodium intake while increasing the contribution of fortifying salt elements. Although these results provide an overview, further study is needed to assess in detail the impact of the added salt mixture concentrations on macro- and micronutrient content.

### 3.3. Effect of the Mineral Fortification on the Distribution Coefficient

Mineral nutrients in table olives are partitioned between the olive pulp and brine. An empirical (pseudo) distribution coefficient, *K_d_*, was intended to quantify this distribution. This distribution coefficient (*K_d_*) reflects the balance of each element’s concentration between the pulp and the brine once equilibrium is achieved. This calculation is particularly relevant for added elements; microelements in the brine were below detection limits (Table 4).

When estimated considering the equilibrium between the olive pulp and brine (Table 5), *K_d_* values were found to be below 1 for Na, K, and Mg, signifying that these elements were more concentrated in the brines than in the pulp, while values above 1 for Ca and P showed a greater association of these elements with olive pulp components. Variations in *K_d_* values among treatments suggest that packaging traditional *Aloreña de Málaga* (and natural olives in brine in general) in chloride salt mixtures affects mineral distribution. Statistically significant models (*p* ≤ 0.05) with no significant lack of fit (*p* > 0.05) were deduced for K, Ca, and Mg, with the most relevant changes in divalent nutrients. All models indicated interactions between the added salts and are expressed in terms of real components.

The model for potassium was a special cubic, with an adjusted R-squared of 0.7917 and a precision of 7.82, and it took the following form:

*K_dK_* = 0.912 · [KCl] + 1.494 · [CaCl_2_] + 1.5607 · [MgCl_2_] − 1.768 · [KCl] · [CaCl_2_] − 1.878 · [KCl] · [MgCl_2_] − 3.584 · [CaCl_2_] · [MgCl_2_] + 5.994 · [KCl] · [ CaCl_2_] · [MgCl_2_] (2)

The model for calcium was quadratic, with an adjusted R-square of 0.9233 and a precision of 17.36, and it took the following form:

*K_dCa_* = 2.334 · [KCl] + 5.85 · [CaCl_2_] + 1.82 · [MgCl_2_] − 10.46 · [KCl] · [CaCl_2_] − 9.07 · [CaCl_2_] · [MgCl_2_] (3)

In the case of magnesium, the model had an adjusted R-squared of 0.47 and a precision of 7.14, taking the following form:

*K_dMg_* = 1.01 · [KCl] + 0.37 · [CaCl_2_] + 0.38 · [MgCl_2_] + 1.67 · [CaCl_2_] · [MgCl_2_] (4)

**Figure 1:**
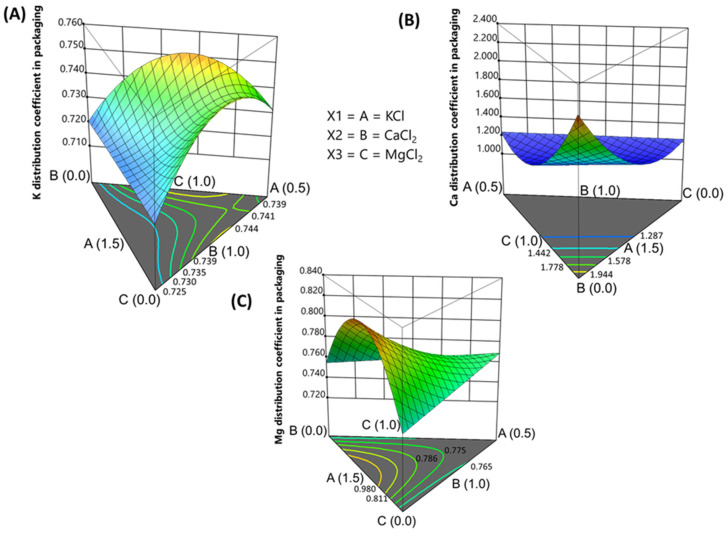
Fortification of cracked green traditional *Aloreña de Málaga* table olives with KCl, CaCl_2_, and MgCl_2_ mixtures during packaging. Evolution in the pseudo distribution coefficient (*K_d_*) in response to KCl, CaCl_2_, and MgCl_2_ concentrations in the packaging brine. (**A**) *K_dK_*; (**B**) *K_dCa_*; and (**C**) *K_dMg_*.

Interpreting these equations becomes more intuitive when viewed as a simplex plot (Figure 1). Generally, changes in *K_d_* were moderate. For potassium, *K_d_* exhibited a curved surface, with a maximum at intermediate KCl concentrations. *K_d_* decreased both at the highest KCl concentration and as the KCl proportion decreased, with the *K_d_* minimum occurring at half-levels of CaCl_2_ and MgCl_2_ proportion, and the KCl was the highest level. For calcium, the changes were well characterised. The *K_dCa_* ranged from 1.2 at 1% to about 0.5% CaCl_2_ concentrations, increasing above 2 without Ca (CaCl_2_ vertex). This suggests that, in the absence of added Ca, the *K_dCa_* is at a maximum because the natural and incorporated Ca is quickly absorbed and firmly bound to the olive pulp components while scarcely present in the brine.

Conversely, above 0.5% CaCl_2_, bounding sites in the pulp may be saturated, and the excess Ca is distributed between the brine and the olive moisture. For magnesium, the highest *K_dMg_* occurred at approximately half the concentrations of CaCl_2_ and MgCl_2_, along with the maximum KCl levels. This indicates that brine’s potassium drives magnesium and calcium into olive pulp. However, increasing CaCl_2_ and MgCl_2_ concentrations decrease the *K_d_* value due to a saturation effect in the pulp, with both elements tending to remain in the brine. The effect was less pronounced at lower K levels. The decrease in *K_d_* with excess Ca and Mg suggests these elements have a strong binding capacity with the olive components. As a result, adding Ca beyond the pulp’s absorption capacity is ineffective, as it will only equilibrate between the olive moisture and brine without being incorporated into the pulp.

Traditionally, mineral elements have been assumed to equilibrate solely to moisture when calculating Na concentrations. Since *Aloreña de Málaga* olives are not lye-treated, it is important to estimate the moisture content across the diverse treatments and assess how it is affected by the salt mixtures. The average moisture content for the olives involved in the experiment was 65.158 g/100 g, ranging from 64.565% to 65.852%. Although the effect of treatments was relatively small (1.287%), it could still be of technological interest. Moisture content was significantly related to the salt mixtures (adjusted R-squared, 0.6961; precision 5.98) and followed the following form, expressed in terms of the actual components:

Moisture (%) = 19.18 · [KCl] + 15.65 · [CaCl_2_] + 10.39 · [MgCl_2_] + 13.64 · [KCl] · [CaCl_2_] + 17.60 · [KCl] · [MgCl_2_] + 21.45 · [CaCl_2_] · [MgCl_2_] − 15.38 · [KCl] · [CaCl_2_] · [MgCl_2_] (5)

**Figure 2:**
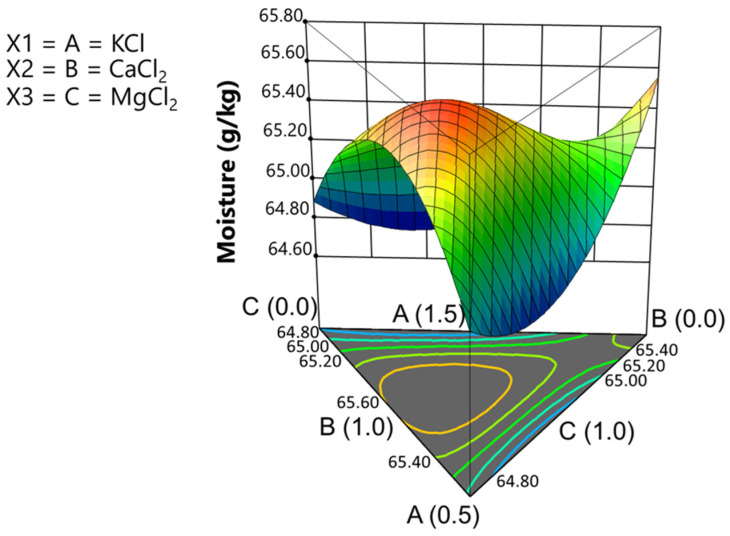
Fortification of cracked green traditional *Aloreña de Málaga* table olives with KCl, CaCl_2_, and MgCl_2_ mixtures during packaging. Graph of the moisture model based on varying concentrations of KCl, CaCl_2_, and MgCl_2_ in the packaging brine.

The equation illustrates the multiple factors influencing moisture content in natural olives. From the plot in the simplex (Figure 2), it can be inferred that the maximum moisture occurs near the barycentre of the plot, at approximately half the range of the contributions of KCl and MgCl_2_ but around 2/3 of the maximum calcium. Variability in olive pulp moisture is a typical controversy in the table olive industry. This study demonstrates that moisture content is influenced by the chloride salts used in this experiment. As a result of the observed changes, the mineral contents, as a function of olive moisture, were estimated using the specific moisture values of each treatment.

When the distribution coefficient (*K_d_*) was calculated, assuming minerals were solely dissolved in the pulp moisture (Table 5), Na values were approximately 1, suggesting that concentrations between the pulp moisture and the brine were nearly at equilibrium. The *K_d_* figures for K and Mg were slightly above 1, suggesting that assuming these nutrients are present exclusively in the moisture would result in overestimating their concentrations relative to the brine. This could be more reasonable, considering that some of these nutrients are also associated with olive components. For calcium and phosphorous, the *K_d_* values indicate that assuming their presence only in pulp moisture would lead to unrealistic high concentrations. Therefore, a sensible portion of their content is likely associated with the olive pulp (as reflected by the high *K_d_* values in Table 5).

### 3.4. Insights into the Distribution of the Mineral Nutrients in the Olive Pulp

The relationship between nutrient minerals in olive pulp (or its moisture) and the brine in fermented green Spanish-style olives was studied through regression analysis.

Applying this approach to non-lye-treated packaged olives with salt mixtures remains unexplored, mainly because data on the mineral equilibrium in the natural table olives is limited. Extending this analysis to these final products poses specific challenges. If the model achieves significance, the *β*-value would represent the mineral’s influence from the brine. The intercept signifies the initial excess or shortage of the mineral in the pulp (or moisture) relative to the brine. The slope, in turn, illustrates the relationship between the concentrations in both substrates. This study, therefore, extends the *K_d_* information by offering insights into the variables influencing the mineral behaviours derived from the above-commented results. It clarifies if the mineral concentrations in the pulp are caused by reactions with olive components or merely the dissolution in pulp moisture. Furthermore, no information is currently available on Mg distribution in table olives.

Among the regression models relating mineral content in the pulp and moisture against brine, those for Na and the added nutrients (K, Ca, and Mg) were analysed (Table 6). Such equations resulted in a high explained variance, with adjusted r-squared values ranging from 0.9729 to 0.9978, suggesting that they accounted for a large portion of the variance (Table 6). The *β* coefficients, reflecting the contribution of brine minerals to pulp concentrations, were also high (0.9866 to 0.9987), showing that their presence in brine significantly impacted the concentrations within the pulp (Table 6). Nevertheless, the model parameters varied depending on the specific mineral and whether pulp or moisture contents were used as the dependent variables.

The intercepts for Na in both pulp and moisture were negative, indicating a non-significant (*p*-values > 0.05) sodium deficiency in the pulp beyond equilibrium expectations, suggesting that Na concentrations in pulp or moisture were lower than anticipated. This implies that some Na was irreversibly removed from olives during desalting. In contrast, potassium showed significant and high intercept values, suggesting a higher content than expected for an equilibrium with added KCl, suggesting a contribution from the olive pulp or moisture independent of the added minerals, aligning with the natural K content in fresh fruits. The Mg intercept was also positive but was non-significant, reflecting a more limited natural contribution to the concentration found in the pulp. Calcium showed the most notable difference among nutrients, with highly significant and positive intercepts, indicating a higher concentration in the pulp than expected from equilibrium with brines, implying a substantial natural contribution from the olives.

The slopes for Na, K, and Mg in the pulp versus the brine were below 1, indicating that, under these conditions, the added minerals tended to increase their concentrations preferentially in the brines over those in the pulp. On the contrary, the calcium slope was much greater compared to those for Na, K, and Mg, suggesting that the Ca addition tends to accumulate the element in the pulp relative to the brine. When the regressions were calculated considering moisture, the slopes for Na and K were close to 1, while that for Mg was slightly higher, and the slope for Ca was the highest at 1.73. This suggests that aside from the deficit in minerals (in the case of Na) or the excess of K (due to the olives’ natural contribution), an equilibrium for these elements is primarily established between olive moisture and brine. However, the slopes for Mg and especially Ca, being above 1, indicate that considering only their presence in olive moisture would lead to unrealistic values. Therefore, some of these minerals are likely absorbed into the olive pulp, forming complexes with its components.

The comparison between both regressions showed that desalting lowers Na levels in the pulp moisture and eliminates some Na that was initially bound to organic components. This Na is not recovered after packaging. K follows a similar trend. However, Mg (to a lesser extent) and Ca are likely already associated with olive pulp components and are less affected by desalting than Na. When added, these minerals may bind to available receptor sites in the pulp, which can absorb more cations. Further additions increased solubilisation in the moisture, but they primarily are bound to reactive sites until saturation is reached.

This builds on the hypothesis of Na equilibrium between pulp moisture and brine, previously explored in fermented green Spanish-style olives, and extends it to packaged natural olives and the minerals K and Mg. It also highlights that a portion of Na lost during desalting (though smaller than in the Spanish-style olives) is not recovered, while Ca remains largely bound to pulp components; although, it may also be present in pulp moisture once the active sites reach saturation.

No models could be constructed for mineral micronutrients, as their concentrations in the brine were below the detection limits of the AA technique used. The minimal changes observed in the pulp of packaged olives compared to desalted ones suggest that these residual micronutrients remain firmly bound to pulp components, preventing significant leakage after packaging.

### 3.5. Predictions of Mineral Concentrations in Olive Pulp at Equilibrium Based on Experimental Design Levels

As previously commented, sodium was excluded from the design, as its level was controlled in the desalting process and maintained after packaging by adding 2.5% NaCl to brine mixtures. In the experimental treatments, Na content in the pulp averaged 6665 mg/kg, compared to 11,915 mg/kg in the current packaging practices (“Control”), nearly double that in the design fortified runs. This demonstrates that the goal of reducing NaCl in the fortified products was successfully achieved.

Equations were developed, as outlined in the Materials and Methods section, to predict the concentrations of the other minerals in the pulp based on the designed concentrations of KCl, CaCl_2_, and MgCl_2_ in the packaging brines. Only the elements derived from the salts used in the brine were significant predictors and could be relied upon for accurate estimates. The models showed a good fit (*p*-value ≤ 0.05), non-significant lack of fit (*p*-value > 0.05), precision (signal-to-noise ratio > 4), and high explained variance. The specific details of each model will be presented separately. The equations were formulated to represent concentrations in actual units (e.g., mg/kg pulp); for values expressed in mg/100 g, the coefficients for the linear terms should be divided by 10, and those for two-way interactions should be divided by 100.

*Potassium.* The proposed model for this element was quadratic, exhibiting high precision (136) and a high explained variance (adj R^2^ = 0.985). The model had linear coefficients for all salts (KCl, CaCl_2_, and MgCl_2_) and the interaction KCl and CaCl_2._ The equation for predicting the expected potassium content in the pulp was as follows:

K in pulp (mg/kg) = +9301.59 · [KCl] − 735.10 · [CaCl_2_] + 1708.66 · [MgCl_2_] + 4311.66 · [KCl] · [CaCl_2_] (6)

**Figure 3:**
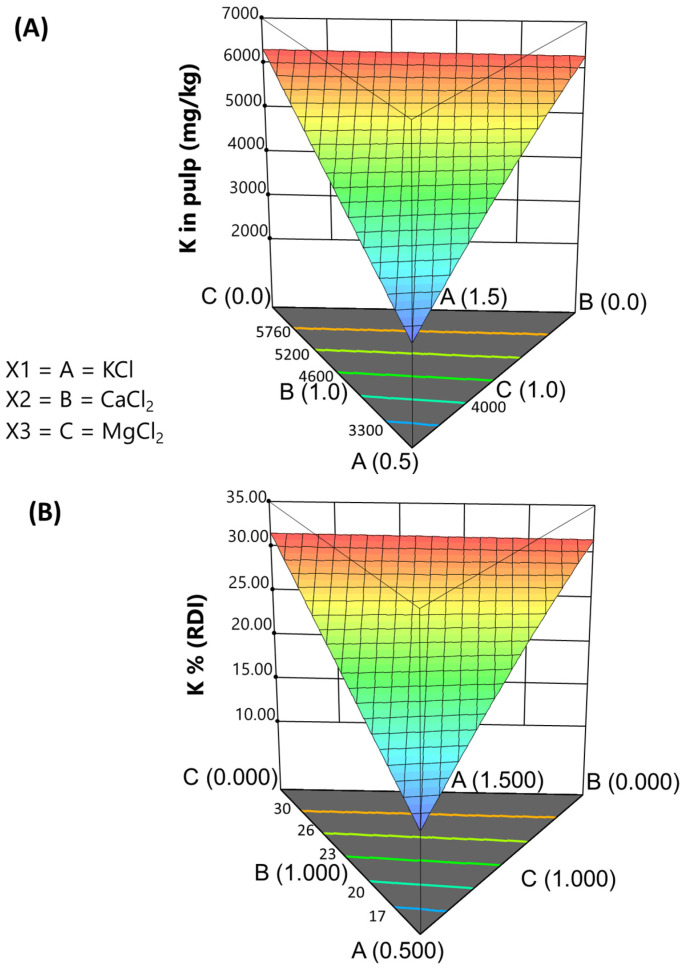
Fortification of cracked green traditional *Aloreña de Málaga* table olives with KCl, CaCl_2_, and MgCl_2_ mixtures during packaging. Relationship between the concentrations of KCl, CaCl_2_, and MgCl_2_ in the packaging brines and the resulting potassium concentrations in the pulp, illustrated as follows: (**A**) expected concentrations in pulp; (**B**) expected contributions (%) to the RDI.

The equation suggests that the potassium content in the pulp is primarily associated with the KCl levels in the packaging brine. Additionally, magnesium contributes to potassium incorporation, while calcium may interfere with K incorporation into the olive pulp. This behaviour could be related to the greater consistency of the olive pulp with Ca, which may hinder K diffusion. This relationship is illustrated in the simplex graph (Figure 3A), where potassium levels increase with increasing KCl concentrations, particularly in the CaCl_2_-MgCl_2_ border. The contour lines showing this progression are nearly parallel to the projection of this border onto the base, indicating minimal interaction effects.

*Calcium*. Regarding calcium, the model suggested was also quadratic, demonstrating high precision (202), a high proportion of explained variance (adj, R squared = 0.9995), and a coefficient standard error of 0.26. The equation for predicting Ca concentrations was as follows:

Ca in pulp (mg/kg) = +2203.35 · [KCl] + 9847.61 · [CaCl_2_] + 1037.99 · [MgCl_2_] + 2030.82 · [KCl] · [CaCl_2_] − 3592.81 · [KCl] · [MgCl_2_] + 7013.91 · [CaCl_2_] · [MgCl_2_] (7)

**Figure 4:**
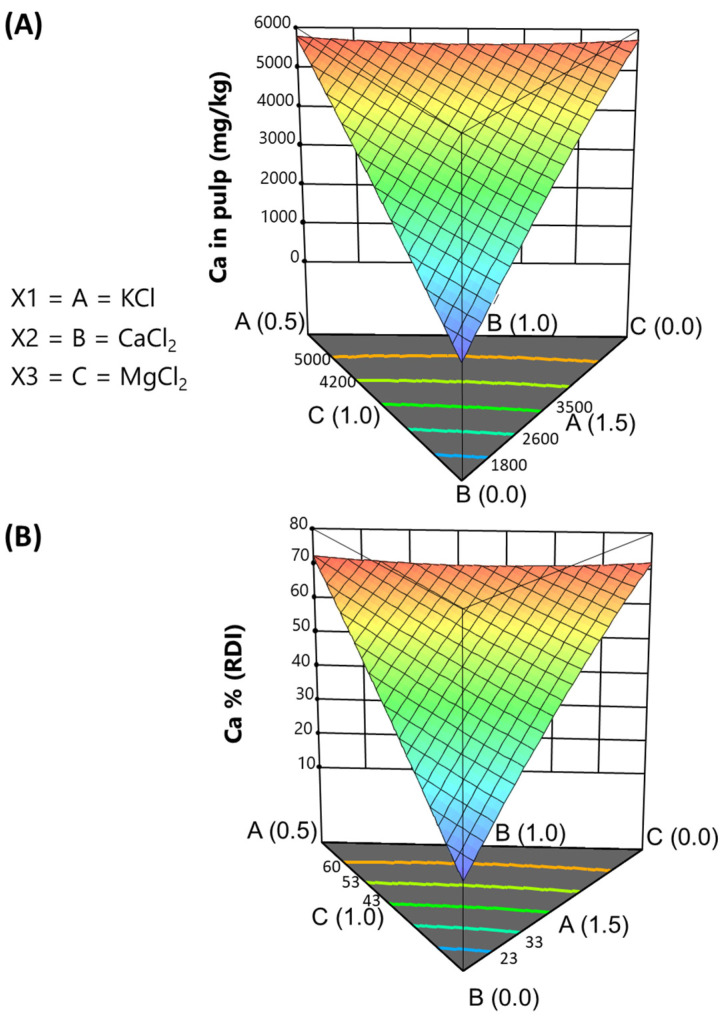
Fortification of cracked green traditional *Aloreña de Málaga* table olives with KCl, CaCl_2_, and MgCl_2_ mixtures during packaging. Relationship between the KCl, CaCl_2_, and MgCl_2_ concentrations in the packaging brines and the resulting calcium concentrations in the pulp, illustrated as follows: (**A**) expected concentrations in pulp; (**B**) expected contributions (%) to the RDI.

The large coefficient for CaCl_2_ signifies a strong association between calcium content in the pulp and the proportion of CaCl_2_ in the packaging brine. Similarly, the positive effects of KCl and MgCl_2_ suggest that these salts also promote Ca absorption into the pulp. Given the equation’s complexity, its interpretation is more effective following its visualisation in the simplex. The plot (Figure 4A) illustrates that Ca content in the pulp rises with higher CaCl_2_ additions, remaining the contour lines nearly parallel to the KCl-MgCl_2_ border. The highest levels of Ca in pulp are also predicted at the border of MgCl_2_-KCl and are almost independent of the proportions of these salts. Theoretically, these concentrations could make olives an interesting source of calcium, as they can lead to sensibly elevated contents.

*Magnesium*. The model for Mg was linear and based solely on the three added salts. It demonstrated high precision (227) and a high adjusted R^2^ (0.9993). The model is as follows:

Mg in pulp (mg/kg) = 18.12 · [KCl] + 56.22 · [CaCl_2_] + 4925.11 · [MgCl_2_] (8)

**Figure 5:**
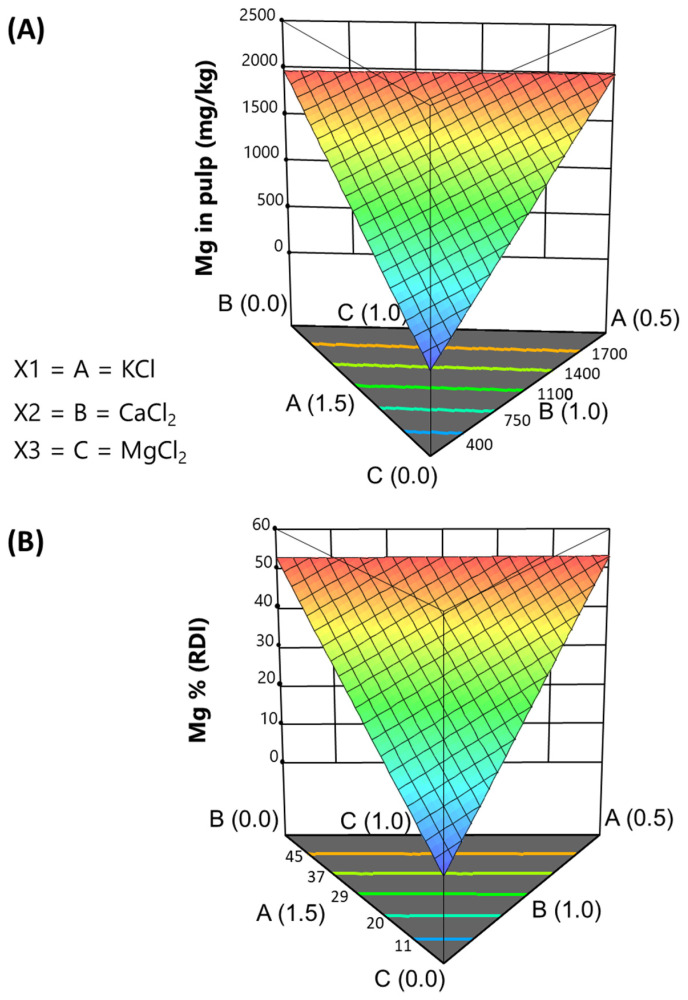
Fortification of cracked green traditional *Aloreña de Málaga* table olives with KCl, CaCl_2_, and MgCl_2_ mixtures during packaging. Relationship between the KCl, CaCl_2_, and MgCl_2_ concentrations in the packaging brines and the resulting magnesium concentrations in the pulp, illustrated as follows: (**A**) expected concentrations in pulp; (**B**) expected contributions (%) to the RDI.

The interpretation of this model is straightforward. It indicates a progressive increase in Mg concentration in the pulp, regardless of the presence of the other components (Figure 5A). The effect reaches its maximum at the CaCl_2_-KCl border, regardless of their respective ratios.

Regarding non-added mineral elements (Table 2), their presence in the packaged olive pulp was unrelated to the levels of the added salts, and their decrease during packaging compared to desalted olives was negligible. In general, the “Control” can exhibit slightly higher contents than the products from the design, indicating that packaging with salt mixtures had minimal impact on these concentrations because of their tough binding to pulp compounds. In the fortified products, Fe concentrations fluctuated between 3.52 and 3.40 mg/kg pulp (versus 3.41 mg/kg in the “Control”), copper content oscillated between 2.23 and 2.17mg/kg pulp (versus 2.21 in the “Control”), zinc ranged from 2.11 to 2.02 mg/kg (versus 2.06 in the “Control”), manganese ranged from 0.335 and 0.292 mg/kg (versus 0.321 in the “Control”), and phosphorous had the highest content, ranging from 75.54 to 73.72 mg/kg (versus 73.13 in the “Control”).

### 3.6. Effect of Fortifying on the Reference Daily Intake (RDI) of Mineral Nutrients

For this study, the Reference Daily Intakes (RDIs) for sodium, potassium, calcium, and magnesium were referenced as follows: 6 g salt or 2300 mg Na/day, 2000 mg K/day, 800 mg Ca/day, and 375 mg Mg/day, respectively. Fortifying in the packaging with KCl, CaCl_2_, and MgCl_2_, with the subsequent 2.5% salt reduction, decreased the Na contribution of these table olives to the RDI by nearly half versus the “Control”, reducing it from 51.80% (11915 mg/kg pulp) to approximately 28.97% (6665 mg/kg pulp). This change had a minimal effect on the concentrations of other naturally present mineral nutrients, with their concentrations in the final experimental products remaining similar to those in the “Control”, including phosphorous, which is the most abundant. Consequently, the effect of fortifying on their contribution to the RDI (%) will not be further commented on.

The estimated RDI contributions of the added salts to the various experimental products are linear combinations of their estimated previous content in the olive pulp. Therefore, the sequential steps for model suggestion and the ANOVA are similar and will not be discussed further. However, their functions differ, and their plots directly illustrate the contributions to the RDI. Both sets of information can be complementary for readers and the table olive industry, enhancing the understanding of mineral contents. The equations and graphs in the simplex for potassium, calcium, and magnesium will be commented on as producers are interested not only in the resulting amounts of the added salts but also in their contributions to the RDI. Proportions exceeding established limits may permit various claims.

*Potassium*. The model predicting the contribution of the salt mixtures used in packaging green natural *Aloreña de Málaga* table olives to the percentage of RDI for potassium is as follows:

%RDI (K) = +46.51 · [KCl] − 3.67 · [CaCl_2_] + 8.54 · [MgCl_2_] + 21.56 · [KCl] · [CaCl_2_] (9)

In the simplex plot (Figure 3B), the range of potassium concentrations applied in this design indicates that contributions can reach around 35% of the RDI, particularly near the CaCl_2_-MgCl_2_ boundary. This finding may potentially back health claims associated with potassium intake.

*Calcium*. The function to predict calcium contribution is as follows:

%RDI (Ca) = +27.54 · [KCl] + 123.10 · [CaCl_2_] + 12.97 · [MgCl_2_] + 25.39 [KCl] · [CaCl_2_] − 44.91 · [KCl] · [MgCl_2_] + 87.67 · [CaCl_2_] · [MgCl_2_] (10)

The graph (Figure 4B) suggests contributions to RDI covering a broad spectrum, increasing with calcium addition up to 70% (at the MgCl_2_-KCl boundary). Given the naturally high Ca concentration in olives, a moderate fortification with CaCl_2_ could surpass the 15% needed to classify traditional cracked green *Aloreña de Málaga* table olives as a source of Ca. This function and its plot confirm that the naturally high proportion of Ca in olives means that a modest addition of CaCl_2_ can yield a calcium-rich product. This information is interesting, as high Ca concentrations are often associated with bitter sensory characteristics.

*Magnesium*. The magnesium contribution to its RDI was predicted by an equation with a similar structure to that derived for Mg content. It is as follows:

%RDI (Mg) = 0.48 · [KCl] + 1.50 · [CaCl_2_] + 131.34 · [MgCl_2_] (11)

The plot (Figure 5B) illustrates extreme values at the CaCl_2_-KCl boundary, with contribution increasing in parallel with the concentrations of KCl and CaCl_2_. The model predicts an RDI contribution of up to approximately 50% at the top levels of MgCl_2_. Nevertheless, percentages exceeding 15% can be reached with a relatively low addition of this salt, potentially supporting, without other considerations, health claims regarding the benefits of magnesium.

### 3.7. Multivariate Evaluation of the Mineral Composition of Experimental Treatments

From an industrial standpoint, comparing the mineral profiles of different treatments aids in selecting salt concentrations that are both cost-effective and aligned with specific consumer preferences.

**Figure 6:**
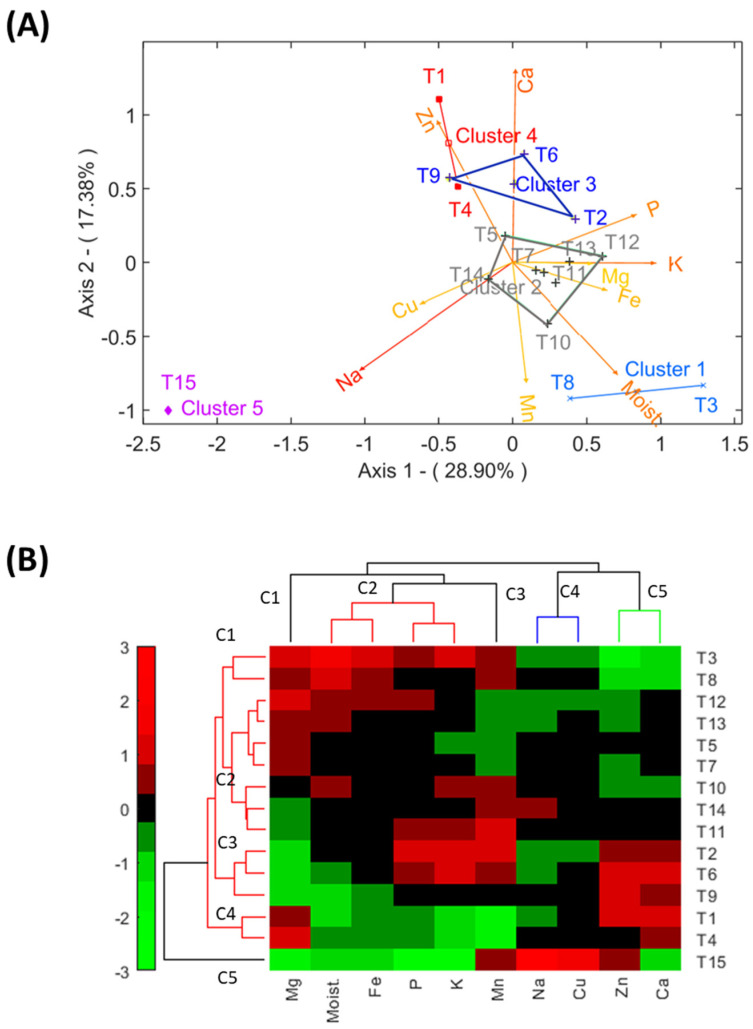
Fortification of cracked green traditional *Aloreña de Málaga* table olives with KCl, CaCl_2_, and MgCl_2_ mixtures during packaging. Grouping the experimental treatments based on their nutrient mineral profiles is shown as follows: (**A**) grouping based on PCA; (**B**) biclustering showing the characteristics of grouped treatments and relationships among mineral contents.

From an industrial standpoint, grouping treatments with similar profiles may aid in choosing salt concentrations that are cost-efficient or meet specific consumer demands. This analysis utilised Principal Components Analysis (PCA) and clustering, focusing on macronutrients (Na, K, Ca, and Mg) and micronutrients (Fe, Cu, Zn, Mn, and P) as well as moisture, which affects the absorption of certain elements (Na, K, or Mg). PCA explained 46.28% of the variance, revealing that treatment 15 (T15), characterised by high Na content, is markedly different from the others (Figure 6A). Sodium, along with potassium and magnesium, influenced differentiation along Axis 1, while calcium (primarily), zinc, manganese, and moisture contributed to differentiation along Axis 2. Treatments 3 (T3) and 8 (T8) were noted for their high moisture, while treatments 1 (T1) and 4 (T4) were associated with elevated levels of calcium and zinc. Additionally, treatments 2 (T2), 6 (T6), and 9 (T9) have relatively elevated levels of calcium and zinc. Treatment 12 (T12) in cluster 2 was linked to potassium and magnesium. Other treatments, located around the centre of the plot, were more challenging to characterise.

Biclustering provides a more precise grouping of treatments (Figure 6B). Treatment 15 (T15) shows great dissimilarity with respect to the experimental treatments. Among the experimental treatments, several groups emerged:

- Cluster 1 (C1) includes treatments 3 (T3) and 8 (T8), which are characterised mainly by high moisture, magnesium (Mg), iron, and potassium but lower levels of zinc (mainly), calcium, copper, and sodium (Figure 6B);
- Cluster 2 (C2) comprises 5 (T5), 7 (T7), 10 (T10), 11 (T11), 12 (T12), 13 (T13), and 14 (T14). These treatments display mineral contents around the average (indicated by the black centre of the plot), with some sporadic higher levels of specific constituents, such as moisture, potassium, and manganese in T10;
- Cluster 3 (C3) includes 2 (T2), 6 (T6), and 9 (T9), which are associated with low magnesium, but high calcium and zinc;
- Cluster 4 (C4) is characterised by high magnesium and calcium.

Despite their cluster assignments, each treatment may also be significant for the presence/absence of specific minerals.

Overall, this multivariate study is a valuable instrument for the olive sector. Manufacturers could choose among several salt mixtures to obtain desired profiles, allowing them to tailor products to meet consumers’ demands, packaging conditions, or product features.

### 3.8. Optimisation

The Design Expert program enables the optimisation of treatments for specific outcomes. Given that the salt contents in the packaging brine need to remain within the established ranges, the goal was to identify a combination of salts that balanced the concentrations of the added or naturally present minerals while maximising the pulp moisture. The specific constraints used in the optimisation process are detailed in Table 7. The optimisation utilised a desirability approach, which assigns scores to responses and chooses factor settings to maximise that score.

The process yielded four solutions (Table 8) featuring KCl, CaCl_2_, and MgCl_2_ concentrations, with the suggested levels being 1.397, 0.825, and 0.278, respectively (Table 8).

**Figure 7:**
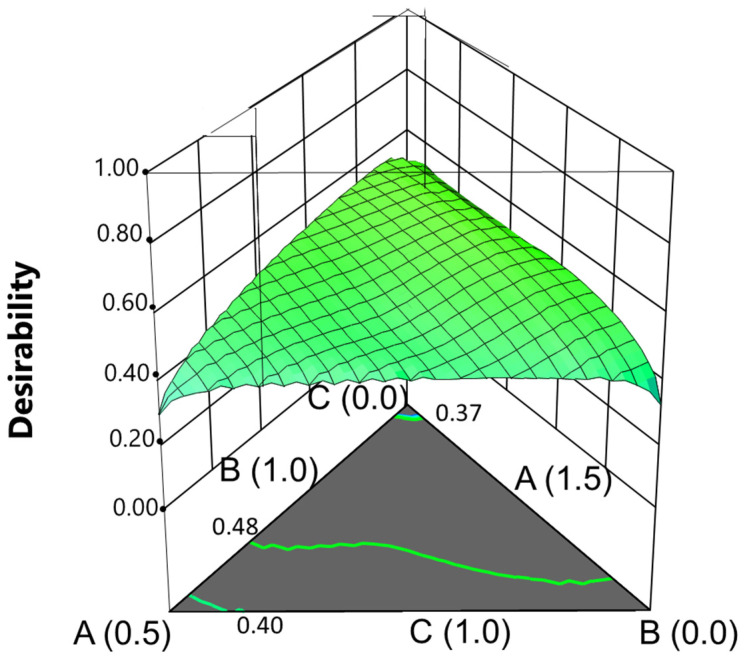
Fortification of cracked green traditional *Aloreña de Málaga* table olives with KCl, CaCl_2_, and MgCl_2_ mixtures during packaging. Graphical optimisation based on the models developed for estimating the mineral concentration in the pulp, based on desirability. Constraints: KCl, CaCl_2_, and MgCl_2_ levels within the range; moisture maximum; simultaneous maximum concentrations of K, Ca, and Mg in the pulp.

Graphically, these coordinates correspond to the point indicated in Figure 7, although the surface shows that the range of concentrations could be somewhat broader without sensible loss of desirability. At the suggested point, the desirability value was relatively high (0.571). Under the above-mentioned concentrations, the responses would yield the following potassium, calcium, and magnesium concentrations in pulp: 593.89, 500.45, and 57.72 mg/100 g pulp. A complete list of mineral and moisture predictions can be found in Table 7.

Notably, all products demonstrated improved nutritional value and reduced sodium levels compared to traditional versions, especially in calcium, potassium, and magnesium. Importantly, this represents just one of the alternatives the experimental design supports for creating products with varying mineral contents. This study offers predictive functions for the concentrations of added minerals and their contributions (%) to RDI, which are suitable for any other salt combinations within the ranges used in this study. Notably, this enhancement was achieved without compromising stability, as these products will soon be progressively stabilised by pasteurisation. Consequently, this study opens a promising line for future research.

## 4. Discussion

Foods like pickles, olives, and sauerkraut are often the first to be eliminated from the diet of patients with high blood pressure or heart failure, who are recommended to reduce their salt intake to 2500–3000 mg/day [25,26]. This guidance often overlooks the nutritional benefits of these foods, which can be rich in monounsaturated fatty acids, polyphenols, vitamin E, and fibre, particularly in cultivars like *Aloreña de Málaga* [5]. Natural brined olives typically retain their pulp better and absorb less sodium than lye-treated ones and represent numerous well-recognised commercial presentations. However, they are usually proscribed to a large proportion of the population. This study demonstrates that these foods can be packaged in chloride salt mixtures, enabling fortified table olives to contain essential minerals like potassium, calcium, and magnesium while significantly reducing salt levels.

Trumbo et al. [27] outlined the challenges and strategies the food industry faces in reducing sodium content, noting the limited availability of low-sodium options for large foodservice operators. In countries with a tradition of table olive consumption, a 50% reduction in sodium could significantly lower overall salt intake. Moreover, natural olives are particularly relevant in this context, as they typically absorb fewer minerals due to their more intact pulp structure [2]. However, modifying traditional processing methods can lead to unexpected outcomes and safety risks. This study examined how K, Ca, and Mg salt mixtures affect mineral distribution between the pulp and brine, using two complementary tools: the pseudo distribution coefficient and regression analysis of mineral contents in pulp (or pulp moisture) compared to brines. Notably, despite the absence of lye treatment, calcium demonstrated a strong affinity for pulp components, indicated by high distribution coefficients (*K_d_*) for both pulp and moisture. Regression analysis revealed high intercept values associated with high calcium levels in the pulp. The pulp and moisture vs brine slopes were unexpectedly steep, especially when using concentrations in moisture. Adding magnesium, another divalent mineral, to the brines also showed some affinity for pulp components, with high intercepts and slopes, though their levels were sensibly inferior than those for calcium. In contrast, sodium and potassium displayed non-significant intercepts and slopes close to 1 (equilibrium) in regressions of pulp moisture versus brines. While altering the usual balance of electrolytes in brines had minimal impact on *K_d_*, the models for the added salts as a function of brine concentrations were still significant.

Fortifying minerals, as a tool for salt replacement during packaging, can lower the salt level in the final product, but it may also remove other minerals initially present in the pulp [22]. This effect is particularly significant for minerals weakly associated with pulp, such as sodium or potassium, while it has minimal impact on micronutrients like iron, copper, zinc, or manganese. Thus, the desalting process for natural olives primarily reduces the loss of beneficial nutritional minerals. The final product contains approximately 44% less sodium and shows enhanced levels of potassium (2578–6349 mg/kg pulp, 10–35% RDI), calcium (858–5801 mg/kg pulp, 10–70% RDI), and magnesium (41–2010 mg/kg pulp, 5–55% RDI). The developed models enable the preparation of specific combinations of salts to achieve targeted mineral profiles and facilitate industry decisions based on consumer demands, requirements, or cost considerations. An overall optimal combination was also included, focusing on maximising concentrations of potassium, calcium, magnesium, naturally present microelements (including phosphorous), and moisture in the pulp.

The health benefits of mineral-fortified olives should be emphasised. The *Dietary Guidelines for Americans 2010 Advisory Committee* identified potassium as a shortfall nutrient, citing evidence linking intake to reduced blood pressure in adults, which lowers the risk of strokes and coronary heart disease. Potassium may also protect against age-related bone loss and reduce the risk of kidney stones [28]. Calcium intake has been associated with numerous health benefits, such as reduce risk of hypertensive disorders during pregnancy, lower blood pressure (particularly in younger individuals), prevention of osteoporosis and colorectal adenomas, and lower cholesterol levels. Studies have also dispelled concerns about calcium supplementation causing adverse effects such as impairing iron status, formation of kidney stones, or myocardial infarction in older individuals [29]. Magnesium, the second most abundant intracellular divalent cation, is a cofactor in numerous metabolic reactions, including protein synthesis, cellular energy production, reproduction, and DNA/RNA synthesis. It is critical in cardiac excitability, neuromuscular conduction, vasomotor tone, and glucose and insulin metabolism. Magnesium deficiency has been linked to chronic conditions such as headaches, Alzheimer’s disease, and cerebrovascular accidents [30]. Given these benefits, incorporating adequate sources of potassium, calcium, and magnesium into the diet is essential for promoting human health.

Interest in mineral fortifying and salt reduction in foods is well established. For instance, Nielsen and Zeuthen [31] investigated the microbiological effects of partially or completely replacing sodium chloride with other cations in model systems. Tassou, Panagou, and Katsaboxakis [32] examined the microbiological changes in naturally fermented *Conservolea* black olives at 4%, 6%, and 8% NaCl. They found that 4% and 6% NaCl promoted lactic acid bacteria growth, resulting in lactic fermentations, while 8% NaCl increased yeast activity, leading to lower final titratable acidity and higher pH.

Kanavouras, Gazouli, Leonidas, and Petrakis [33] compared traditional processes using 16% NaCl with two buffered solutions containing 12% NaCl and different calcium sources. They discovered that a buffer of acetic acid (0.05 M) with Ca(OH)_2_ (0.025 M) and 12% NaCl yielded a product with notably higher texture, colour, and acceptability. In further research on Greek *Conservolea* olives, Tassou et al. [34] found that 4% calcium chloride increased the depth of the peripheral region where cell wall breakage was observed. Higher CaCl_2_ led to skin stiffening.

Hurtado et al. [35] processed *Arbequina* table olives as naturally green olives at different maturation stages and NaCl levels, revealing that green olives delayed lactic acid bacterial growth, while higher NaCl levels favoured the elimination of Enterobacteriaceae and hindered the yeast growth. Panagou et al. [16] tested five different NaCl, KCl, and CaCl_2_ combinations during *Conservolea* natural olives processing, noting that increasing CaCl_2_ content or a combination of KCl and CaCl_2_ imparted bitterness to the product, which became less acceptable. Only a mixture of 4% NaCl and 4% CaCl_2_ produced olives with reduced salt and good acceptability.

Saudé et al. [36] evaluated the effect of chloride salt mixtures (NaCl, KCl, and CaCl_2_) during the fermentation of *Mançanilha Algarvia* cracked olives, reporting optimal results in terms of overall attributes at 8% or 4%NaCl + 4%KCl. Zinno, Guantario, Perozzi, Pastore, and Devirgiliis [37] used brines with 25%, 50%, and 75% of NaCl substituted with KCl to ferment *Nocellara del Belice* according to *Castelvetrano* methods (7% total salts). Their data indicated that partial NaCl replacement with KCl did not increase contamination risk or the overgrowth of pathogens or spoiler microbes.

Research on the natural *Aloreña de Málaga* table olives is limited. Bautista-Gallego, Arroyo-López, López-López, and Garrido-Fernández [38] studied their fermentation in sodium, potassium, and calcium chloride salt mixtures, finding the best overall quality with 3.7% NaCl and 7.3% KCl, although optimum firmness was around the composition barycentre. As observed, most studies on using other chloride salts in natural (directly brined) olives focused exclusively on sodium replacement during fermentation, and high total salt levels were required to prevent spoilage or pathogens.

Most studies on salt substitution during fermentation have focused on process parameters, with only a few addressing the resulting mineral content. For example, fermenting green Spanish-style *Gordal* olives yielded potassium and calcium levels in the fermented product ranging from about 180 to 900 mg/kg pulp [14]. Similarly, *Manzanilla* olives fermented with brines containing up to 60 g/L of KCl and CaCl_2_ reached significantly higher concentrations, ranging from 682 to 17,802 mg/kg pulp for potassium and from 573 to 7942 mg/kg pulp for calcium [15]. For seasoned cracked *Aloreña de Malaga* packed with NaCl, KCl, and CaCl_2_, a previous study reported mineral contents ranging from 961 to 5557 mg/kg pulp for potassium and 1031 to 3566 mg/kg pulp for calcium [39]. However, that experiment did not include a desalting step, and magnesium was not considered. In our study, the final mineral content was even higher, ranging from 2596 to 6280 mg/kg pulp for potassium and 858 to 5801 mg/kg pulp for calcium.

The impact of potassium and calcium fortification on the sensory characteristics of fermented products has been widely studied. In green Spanish-style *Gordal* fermentations, bitterness, an undesirable attribute, was directly associated with calcium content. However, calcium also improved desirable attributes like hardness and crunchiness [14]. A similar relationship between bitterness and calcium was observed in green Spanish-style *Manzanilla* olives [15]. For naturally fermented *Aloreña de Malaga* olives, bitterness was also linked to calcium, though the association with crunchiness or fibrousness was weaker [39]. In natural black *Gemlik* olives, combinations of KCl and CaCl_2_ salts increased bitter taste, but a mixture of 5% NaCl and 5% KCl resulted in good sensory qualities [40]. Conversely, in *Castelvetrano* olives, substituting NaCl with KCl intensified bitterness in both Spanish-style and local processing, although to levels within product acceptance [17]. Similarly, in the Greek naturally black *Coservolea* olives, partial substitution of NaCl with KCl and CaCl_2_ yielded a bitter product with low acceptability, but a mixture of 5% NaCl and 5% KCl received good acceptation [16]. In plain Spanish-style *Manzanilla* olives packaged with mixtures of KCl, CaCl_2_, and MgCl_2_, the lowest bitter scores were achieved with maximum KCl concentrations (1.5%) and approximately half the range of CaCl_2_ and MgCl_2_ levels [41]. Then, the effects of potassium, calcium, and magnesium chloride salts on bitter notes remain complex and require further investigation. Nevertheless, acceptable combinations with favourable organoleptic characteristics can be identified, and the RSM has proven to be an effective tool for optimising formulations.

As highlighted above, fortifying/substituting sodium chloride with potassium, calcium, and magnesium chloride salts can produce table olives with enhanced levels of essential mineral nutrients and acceptable sensory characteristics. When these salts are added during packaging, the resulting olives can exhibit reduced bitter tones and greater consumer acceptability. Although the production costs of these fortified products may be slightly higher, they offer a healthier profile, aligning with the current consumer favouring nutritious and health-conscious food choices.

As a result, our study involved a desalting process to control sodium levels in the final products better and included all the macro and micronutrient of interest. This study on *Aloreña de Málaga* olive fortification during packaging is, then, a novel approach for enhancing healthy olive characteristics (K, Ca, and Mg added) and reducing cardiovascular risks (lower Na content). Moreover, this research provides new insights into magnesium, which had not been included in previous combinations. We also examined the distribution of minerals between the brine and pulp or moisture and developed models to predict the expected concentrations of potassium, calcium, and magnesium in the pulp. Additionally, we grouped treatments according to their similarities and established optimal overall concentrations tailored to specific consumer or producer requirements.

## 5. Conclusions

This study builds on a previously developed strategy of replacing salt in fermented/stored products during packaging rather than during fermentation or storage. It applied this approach to natural green traditional *Aloreña de Málaga* table olives, a non-lye-treated product with reduced environmental impact. Plan design and analysis were conducted using RSM.

By incorporating KCl, CaCl_2_, and MgCl_2_ into the packaging brines, we maintained a reasonable sodium level while enhancing nutritional characteristics and promoting eco-friendly natural products. Since these olives are not lye-treated, they better retain their original nutrients, further enhanced by the added elements.

This study revealed that the added salts significantly influenced some minerals’ distribution between olive pulp and brine. As seen in Spanish-style olives, sodium and potassium are primarily distributed between the brine and olive moisture, whereas Mg and Ca (primarily) are predominantly bound to pulp components. Micronutrients such as iron, copper, zinc, and manganese are also strongly linked to olive pulp components. Initial P can be released into the desalting solution and packaging brine, but its content in the final product is still noticeable.

Potassium, calcium, and magnesium contents increased significantly: potassium rose from 657 mg/kg pulp to between 2578 and 6349 mg/kg pulp, calcium increased from 858 mg/kg pulp to between 858 and 5801 mg/kg pulp, and magnesium grew from 41 mg/kg pulp to between 41 and 2010 mg/kg pulp. In contrast, sodium concentrations decreased from 11,915 mg/kg in the traditional product to 6665 mg/kg pulp in the modified olives. This indicates that these olives can contribute to the daily K, Ca, and Mg intakes by 10–35%, 10–70%, and 5–55% of the Reference Daily Intake (RDI), respectively, while that of sodium decreased from 52% to 29%.

The developed functions enable the prediction of mineral contents and contributions (%) to the RDI for any combination of salts, allowing for optimisation based on specific constraints. Additionally, multivariable analysis visually associated the different treatments with the added minerals, grouping them into categories to help select the most suitable options.

Overall, the changes in mineral composition observed in the experimental treatments position them as a valuable source of essential nutrients. This information can assist industries in developing new products with healthy mineral profiles that comply with the WHO and EU guidelines for reducing sodium intake while increasing essential minerals such as potassium, calcium, and magnesium. Additionally, it may help address the potassium deficiency recently identified in the EU population, as highlighted in the *Scientific advice related to nutrient profiling for the development of harmonised mandatory front-of-pack nutrition labelling and the setting of nutrient profiles for restricting nutrition and health claims on foods* [42].

## Figures and Tables

**Table 1:** Fortification of cracked green traditional *Aloreña de Málaga* table olives with KCl, CaCl_2_, and MgCl_2_ mixtures during packaging. Lattice mixture experimental design used for the experiment.

Treatment	NaCl	KCl	CaCl_2_	MgCl_2_
1	2.50	0.50	1.00	1.00
2	2.50	1.50	1.00	0.00
3	2.50	1.50	0.00	1.00
4	2.50	0.50	1.00	1.00
5	2.50	1.00	0.50	1.00
6	2.50	1.50	0.50	0.50
7	2.50	0.83	0.83	0.83
8	2.50	1.50	0.00	1.00
9	2.50	1.50	1.00	0.00
10	2.50	1.33	0.33	0.83
11	2.50	1.33	0.83	0.33
12	2.50	1.17	0.67	0.67
13	2.50	1.00	0.50	1.00
14	2.50	1.00	1.00	0.50
15 (Control)	5.00	0.00	0.00	0.00

Note: The actual concentrations in the packaging brine were readjusted considering the ratio of olive/brine in containers, moisture in the pulp, and the degree of hydration of the salts (KCl, CaCl_2_·2H_2_O, and MgCl_2_·6H_2_O).

**Table 2:** Fortification of cracked green traditional *Aloreña de Málaga* table olives with KCl, CaCl_2_, and MgCl_2_ mixtures during packaging. Mineral characteristics of the stored fruits and the desalted product (the material to be packaged).

Element	Stored Olives				Desalted Olives (Raw Material)		
	Pulp		Brine		Pulp		Released Mineral (%)
	Mean	SE	Mean	SE	Mean	SE	
Na (mg/kg)	18590	223	29523	95	7205	4	61.24
K (mg/kg)	2468	11	3987	2	843	1	65.84
Ca (mg/kg)	1189	12	708	1	1035	1	12.95
Mg (mg/kg)	126.4	0.7	134.1	0.4	57	1	54.91
P (mg/kg)	189	2	213	1	119	(<1)	37.04
Fe (mg/kg)	5.55	0.04	1.6	0.06	4.04	0.03	27.21
Cu (mg/kg)	3.31	0.03	0.45	0.01	2.65	0.04	19.94
Mn (mg/kg)	1.04	0.06	1.2	0.01	0.52	0.01	50.00
Zn (mg/kg)	4.03	0.03	1.14	0.01	2.57	0.02	36.23
Moisture	61.24	0.09			66.62	0.13	−8.79

Notes: The negative sign for moisture should be interpreted as moisture increases, 100 × ((initial − final values)/initial value).

**Table 3:** Fortification of cracked green traditional *Aloreña de Málaga* table olives with KCl, CaCl_2_, and MgCl_2_ mixtures during packaging. Mineral nutrients in the pulp (mg/kg) of the packaged product. The table also includes the percentages of moisture in pulp necessary for some calculations.

Treatment	Na	K	Ca	Mg	Fe	Cu	Mn	Zn	P	Moisture (%. *w*/*w*)
1	6692.47 (63.67)	2595.97 (36.35)	5789.30 (17.62)	2010.78 (21.14)	3.40 (<0.01)	2.18 (0.03)	0.292 (0.027)	2.09 (0.02)	74.27 (0.79)	64.791 (0.088)
2	6679.21 (89.41)	6280.38 (25.06)	5710.34 (14.66)	41.01 (1.34)	3.47 (0.05)	2.17 (<0.01)	0.330 (0.015)	2.04 (0.02)	74.98 (0.62)	64.817 (0.048)
3	6688.13 (53.81)	6274.78 (17.66)	903.91 (22.80)	1979.05 (16.99)	3.52 (0.05)	2.19 (0.03)	0.316 (0.011)	2.03 (0.01)	75.42 (0.63)	65.691 (0.128)
4	6686.75 (56.53)	2578.12 (12.69)	5801.31 (13.27)	1985.49 (21.98)	3.44 (0.04)	2.21 (0.04)	0.291 (0.015)	2.07 (0.04)	73.92 (1.43)	65.287 (0.068)
5	6684.71 (83.60)	4648.06 (19.30)	3408.78 (51.32)	1989.36 (25.20)	3.45 (0.03)	2.21 (0.02)	0.299 (0.031)	2.04 (0.05)	74.46 (0.49)	64.739 (0.089)
6	6667.79 (32.37)	6265.73 (10.66)	3567.79 (39.99)	1009.84 (30.87)	3.41 (0.04)	2.18 (0.03)	0.324 (0.020)	2.11 (0.01)	75.54 (0.47)	64.778 (0.130)
7	6636.34 (66.86)	3818.71 (19.49)	4968.20 (53.97)	1662.21 (5.46)	3.43 (0.03)	2.19 (0.02)	0.313 (0.021)	2.02 (0.04)	74.44 (0.55)	65.251 (0.086)
8	6646.32 (71.23)	6269.07 (23.28)	858.58 (21.04)	2007.44 (6.73)	3.42 (0.03)	2.19 (0.03)	0.324 (0.010)	2.02 (0.03)	73.90 (0.79)	65.420 (0.099)
9	6650.54 (51.54)	6349.02 (56.88)	5789.88 (34.00)	41.85 (1.66)	3.49 (0.04)	2.23 (0.02)	0.299 (0.024)	2.08 (0.01)	73.72 (0.76)	64.934 (0.139)
10	6629.32 (45.91)	5732.91 (14.84)	2631.93 (10.32)	1613.50 (15.03)	3.45 (0.04)	2.19 (0.02)	0.328 (0.007)	2.06 (0.01)	73.87 (0.41)	65.346 (0.116)
11	6664.16 (58.75)	5700.70 (78.72)	5021.57 (10.06)	673.85 (4.77)	3.41 (0.03)	2.21 (0.02)	0.329 (0.009)	2.04 (<0.01)	75.42 (0.27)	65.445 (0.136)
12	6659.21 (70.51)	5115.82 (13.66)	4302.01 (19.31)	1327.05 (13.29)	3.49 (0.04)	2.18 (0.01)	0.296 (0.027)	2.05 (0.03)	74.25 (1.08)	65.598 (0.028)
13	6662.63 (29.32)	4607.05 (11.49)	3399.27 (6.87)	2007.86 (7.59)	3.43 (0.04)	2.20 (0.02)	0.305 (0.022)	2.03 (0.02)	75.38 (0.70)	65.006 (0.127)
14	6665.04 (53.76)	4570.74 (11.71)	5595.78 (8.84)	1017.80 (13.16)	3.44 (0.05)	2.21 (0.01)	0.335 (0.004)	2.07 (0.03)	73.97 (0.58)	65.514 (0.198)
15 ^a^	11,915.33 (91.04)	657.26 (14.32)	857.82 (1.79)	40.63 (1.16)	3.41 (0.02)	2.21 (0.01)	0.321 (0.023)	2.06 (0.03)	73.13 (0.79)	64.757 (0.064)

Note: Standard error in parenthesis. ^a^ Control.

**Table 4:** Fortification of cracked green traditional *Aloreña de Málaga* table olives with KCl, CaCl_2_, and MgCl_2_ mixtures during packaging. Mineral nutrients in the packaged olives’ brine (mg/kg).

Treatment	Na	K	Ca	Mg	P
1	10,335.41 (157.60)	3560.23 (16.91)	4722.28 (19.40)	2625.77 (17.25)	43.87 (0.79)
2	10,469.28 (221.64)	8765.55 (43.64)	4751.35 (14.95)	54.16 (1.26)	46.09 (0.62)
3	10,270.80 (196.96)	8726.68 (21.15)	403.91 (5.64)	2618.74 (9.13)	45.34 (0.63)
4	10,282.60 (223.55)	3506.01 (6.62)	4716.26 (11.17)	2615.57 (5.20)	45.21 (1.43)
5	10,128.76 (234.92)	6228.35 (13.04)	2732.87 (16.78)	2656.20 (6.88)	43.59 (0.49)
6	10,258.87 (239.57)	8718.03 (36.55)	2661.72 (3.54)	1214.76 (14.53)	44.89 (0.47)
7	10,415.69 (168.12)	5209.98 (19.47)	4102.91 (13.67)	2134.20 (4.70)	44.67 (0.55)
8	10,424.60 (84.11)	8686.84 (6.65)	412.01 (5.38)	2611.49 (26.07)	44.19 (0.79)
9	10,055.74 (160.15)	8782.44 (41.37)	4714.95 (7.54)	58.13 (2.31)	44.34 (0.76)
10	10,117.15 (239.51)	7842.96 (15.93)	2067.19 (21.74)	2121.87 (23.21)	44.04 (0.41)
11	10,189.75 (114.24)	7783.81 (11.33)	4150.31 (6.21)	847.94 (26.60)	43.96 (0.27)
12	10,068.51 (76.22)	6997.38 (22.43)	3335.24 (7.95)	1711.08 (17.69)	46.09 (1.08)
13	10,227.57 (293.35)	6080.57 (9.64)	2599.73 (19.40)	2593.61 (16.06)	44.37 (0.70)
14	10,405.45 (145.64)	6114.78 (18.26)	4660.60 (7.62)	1292.88 (7.27)	44.59 (0.58)
15 ^a^	18,159.13 (99.84)	909.13 (17.42)	436.87 (3.31)	57.79 (0.92)	45.34 (0.79)

Note: Standard error in parenthesis. The contents of Fe, Cu, Mn, and Zn were below detection limits in the brine after packaging. ^a^ Control.

**Table 5:** Fortification of cracked green traditional *Aloreña de Málaga* table olives with KCl, CaCl_2_, and MgCl_2_ mixtures during packaging. Distribution coefficient values (*K_d_*) for the mineral nutrients added and phosphorous.

Treatment	Considering Mineral Distribution in Pulp Versus Brine					Considering Mineral Distribution in Pulp Moisture Versus Brine				
	Na	K	Ca	Mg	P	Na	K	Ca	Mg	P
1	0.648 (0.005)	0.729 (0.014)	1.226 (0.009)	0.766 (0.005)	1.694 (0.035)	1.000 (0.006)	1.126 (0.021)	1.892 (0.012)	1.182 (0.006)	2.615 (0.052)
2	0.639 (0.019)	0.717 (0.003)	1.202 (0.003)	0.757 (0.006)	1.627 (0.021)	0.985 (0.030)	1.105 (0.005)	1.854 (0.010)	1.69 (0.033)	2.510 (0.033)
3	0.652 (0.010)	0.719 (0.001)	2.390 (0.001)	0.756 (0.067)	1.664 (0.023)	0.992 (0.017)	1.095 (0.002)	3.408 (0.102)	1.150 (0.102)	2.532 (0.032)
4	0.651 (0.010)	0.735 (0.003)	1.230 (0.003)	0.759 (0.004)	1.637 (0.031)	0.997 (0.017)	1.126 (0.005)	1.884 (0.006)	1.163 (0.011)	2.507 (0.045)
5	0.660 (0.010)	0.746 (0.005)	1.248 (0.005)	0.749 (0.026)	1.709 (0.040)	1.020 (0.016)	1.153 (0.007)	1.927 (0.043)	1.157 (0.015)	2.640 (0.066)
6	0.651 (0.016)	0.719 (0.004)	1.340 (0.004)	0.831 (0.015)	1.683 (0.010)	1.001 (0.025)	1.110 (0.008)	2.069 (0.027)	1.128 (0.026)	2.598 (0.021)
7	0.638 (0.013)	0.733 (0.002)	1.211 (0.002)	0.779 (0.014)	1.667 (0.015)	0.977 (0.021)	1.123 (0.004)	1.856 (0.024)	1.194 (0.005)	2.555 (0.023)
8	0.638 (0.009)	0.722 (0.002)	2.084 (0.002)	0.769 (0.036)	1.672 (0.007)	0.975 (0.014)	1.103 (0.004)	3.185 (0.053)	1.175 (0.010)	2.557 (0.008)
9	0.662 (0.015)	0.723 (0.010)	1.228 (0.010)	0.724 (0.006)	1.664 (0.034)	1.019 (0.023)	1.113 (0.014)	1.891 (0.009)	1.115 (0.085)	2.562 (0.054)
10	0.656 (0.014)	0.731 (0.001)	1.274 (0.001)	0.760 (0.002)	1.678 (0.024)	1.003 (0.022)	1.119 (0.001)	1.949 (0.032)	1.164 (0.004)	2.567 (0.035)
11	0.655 (0.004)	0.732 (0.009)	1.210 (0.009)	0.797 (0.003)	1.716 (0.029)	0.999 (0.007)	1.119 (0.012)	1.849 (0.008)	1.217 (0.048)	2.622 (0.39)
12	0.662 (0.011)	0.731 (0.004)	1.289 (0.004)	0.776 (0.009)	1.613 (0.054)	1.008 (0.017)	1.115 (0.006)	1.966 (0.014)	1.182 (0.004)	2.459 (0.083)
13	0.652 (0.021)	0.758 (0.001)	1.308 (0.001)	0.774 (0.012)	1.700 (0.049)	1.000 (0.034)	1.166 (0.002)	2.012 (0.019)	1.191 (0.010)	2.615 (0.080)
14	0.641 (0.014)	0.748 (0.001)	1.201 (0.001)	0.787 (0.004)	1660 (0.033)	0.970 (0.024)	1.141 (0.005)	1.833 (0.001)	1.202 (0.017)	2.533 (0.044)
15 ^a^	0.656 (0.004)	0.724 (0.026)	1.964 (0.026)	0.703 (0.001)	1.614 (0.037)	1.013 (0.005)	1.118 (0.039)	3.033 (0.021)	1.085 (0.018)	2.493 (0.054)

Note: Standard error in parenthesis. ^a^ Control.

**Table 6:** Fortification of cracked green traditional *Aloreña de Málaga* table olives with KCl, CaCl_2_, and MgCl_2_ mixtures during packaging. Models relating mineral concentrations in pulp or moisture versus contents in brine.

Model/ Summary		Na	K	Ca	Mg
		Concentration in Pulp (mg/kg) vs. Concentration in Brine (mg/kg)			
Full model	Adjusted R^2^	0.9748	0.9971	0.9973	0.9978
	*p*-value	<0.0001	<0.0001	<0.0001	<0.0001
R. summary	β	0.9876	0.9986	0.9987	0.9989
	SE of β	0.0240	0.0082	0.0079	0.0070
	Intercept	−5.4672	93.1697	406.4841	19.1189
	SE	173.1431	40.4284	31.1117	10.3576
	*p*-value	0.9750	0.0261	<0.0001	0.0718
	Slope	0.6508	0.7156	1.1300	0.7583
	SE	0.0158	0.0058	0.0089	0.0053
	*p*-value	<0.0001	<0.0001	<0.0001	<0.0001
		**Concentration in Pulp Moisture (mg/kg) vs. Concentration in Brine (mg/kg)**			
Full model	Adjusted R^2^	0.9729	0.9970	0.9969	0.9977
	*p*-value	<0.0001	<0.0001	<0.0001	<0.0001
R. summary	β	0.9866	0.9985	0.9985	0.9989
	SE of β	0.0248	0.0082	0.0083	0.0072
	Intercept	−156.9743	149.4121	621.9038	27.8193
	SE	279.7584	62.5391	50.4936	16.4187
	*p*-value	0.5776	0.0213	<0.0001	0.0974
	Slope	1.0130	1.0967	1.7356	1.1637
	SE	0.0255	0.0090	0.0145	0.0084
	*p*-value	<0.0001	<0.0001	<0.0001	<0.0001

**Table 7:** Constrains imposed in the overall (desirability) optimisation process.

Name	Goal	Lower Limit	Upper Limit	Importance
A: KCl in design (%, *w*/*w*)	is in range	0.5	1.5	3
B: CaCl_2_ in design	is in range	0	1	3
C: MgCl_2_ in design	is in range	0	1	3
pH	minimise	3.63	3.84	3
Firmness	maximise	33.46	36.09	3
Moisture	maximise	64.7386	65.6906	3
Na in pulp (mg/kg pulp)	minimise	6629.32	6692.47	3
K in pulp (mg/kg pulp)	maximise	2578.12	6349.02	3
Ca in pulp (mg/kg)	maximise	858.575	5801.31	3
Mg in pulp (mg/kg pulp)	maximise	97.0274	98.0212	3
Fe in pulp (mg/kg)	maximise	3.403	3.52133	3
Cu in pulp (mg/kg pulp)	maximise	2.17433	2.22733	3
Zn in pulp (mg/k pulp)	maximise	2.02323	2.11423	3
Mn in pulp (mg/kg pulp)	maximise	0.291	0.334667	3
P in pulp (mg/kg pulp)	maximise	73.7221	75.5392	3
Na contribution RDI (%)	minimise	27.6222	27.8853	3
K contribution RDI (%)	maximise	3.2309	183.246	3
Ca contribution RDI (%)	maximise	0.86158	48.8656	3
Mg contribution RDI (%)	maximise	4.42649	4.54864	3

Note: RDI, Reference Daily Intake.

**Table 8:** Fortification of cracked green traditional *Aloreña de Málaga* table olives with KCl, CaCl_2_, and MgCl_2_ mixtures during packaging. Values expected for the selected parameters of the packaged product at the optimum desirability levels.

Parameter Values	Solution 1	Solution 2	Solution 3	Solution 4
KCl content at equilibrium (g/100 mL)	1.397	1.374	1.500	1.117
CaCl_2_ content at equilibrium (g/100 mL)	0.825	1.000	0.412	0.323
MgCl_2_ content at equilibrium (g/100 mL)	0.278	0.126	0.588	1.000
pH	3.756	3.759	3.756	3.716
Firmness (kN/g pitted olives)	35.526	35.838	34.804	34.554
Moisture in pulp (%, *w*/*w*)	65.154	65.154	64.892	65.004
Na content in pulp (mg/100 g pulp)	666.152	666.226	665.893	666.411
K content in pulp (mg/100 g pulp)	593.896	585.271	628.803	523.127
Ca content in pulp (mg/100 g pulp)	500.449	569.072	315.528	253.307
Mg content in pulp (mg/100 g pulp)	57.720	28.036	117.829	198.583
Fe content in pulp (mg/100 g pulp)	3.455	3.455	3.459	3.448
Cu content in pulp (mg/100 g pulp)	2.198	2.200	2.194	2.192
Zn content in pulp (mg/100 g pulp)	2.073	2.057	2.110	2.029
Mn content in pulp (mg/100 g pulp)	0.320	0.320	0.321	0.312
P content pulp (mg/100 g pulp)	74.610	74.573	74.742	74.618
Na contribution to RDI	27.756	27.759	27.746	27.767
K contribution to RDI	29.695	29.264	31.440	52.956
Ca contribution to RDI	62.656	71.134	39.441	31.654
Mg contribution to RDI	15.392	7.476	31.421	52.956
*K_dNa_*	1.000	0.991	1.006	1.006
*K_dK_*	1.113	1.112	1.112	1.148
*K_dCa_*	1.805	1.893	2.151	2.263
*K_dMg_*	1.209	1.171	1.275	1.168

## Data Availability

The original contributions presented in the study are included in the article, further inquiries can be directed to the corresponding author.
